# Metabolic Reprogramming: A Crucial Contributor to Anticancer Drug Resistance

**DOI:** 10.1002/mco2.70358

**Published:** 2025-09-06

**Authors:** Yunhan Zhu, Weijie Yan, Lingfeng Tong, Jie Yang, Shengfang Ge, Jiayan Fan, Renbing Jia, Xuyang Wen

**Affiliations:** ^1^ Department of Ophthalmology Ninth People's Hospital Affiliated to Shanghai Jiao Tong University School of Medicine Shanghai China; ^2^ Shanghai Key Laboratory of Orbital Diseases and Ocular Oncology Shanghai China; ^3^ State Key Laboratory of Eye Health, Department of Ophthalmology, Shanghai Ninth People's Hospital Shanghai Jiao Tong University School of Medicine Shanghai China; ^4^ Engineering Research Center of Techniques and Instruments for Diagnosis and Treatment of Congenital Heart Disease, Ministry of Education Xinhua Hospital, Shanghai Jiao Tong University School of Medicine Shanghai China

**Keywords:** cancer metabolism, drug resistance, metabolic targeted strategies, tumor microenvironment

## Abstract

Cancer metabolic reprogramming is a fundamental hallmark that enables tumor cells to sustain their malignant behaviors. Beyond its role in supporting growth, invasion, and migration, metabolic rewiring actively contributes to anticancer drug resistance. Cancer cells not only reshape their own metabolism but also engage in aberrant metabolic crosstalk with nonmalignant components within the tumor microenvironment (TME). These metabolic alterations create multiple barriers to the efficacy of drug therapies, including chemotherapy, targeted therapy, and immunotherapy. Despite growing evidence, an integrated understanding of how metabolic reprogramming contributes to the development of drug resistance and how it may be therapeutically targeted to overcome the resistance remains incomplete. This review summarizes recent progresses in tumor‐intrinsic and TME‐associated metabolic alterations that contribute to drug resistance by sustaining metabolic needs and modulating nonmetabolic processes and explores the upstream regulatory mechanisms driving these changes, focusing particularly on glucose, lipid, and amino acid metabolism. We also discuss the current advances in the integration of small molecule inhibitors targeting cancer metabolism to address drug resistance. By consolidating mechanistic insights and therapeutic opportunities, this review highlights metabolic reprogramming as a promising intervention point to overcome anticancer drug resistance.

## Graphical abstract

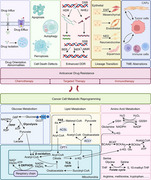

## 1 Introduction

The reprogramming of cancer cell metabolism is considered a primary hallmark of cancer [1]. To support their abnormal survival, proliferation, and migration, cancer cells have high demands for energy resources, biosynthetic materials, and signal transduction‐related molecules. Consequently, they autonomously modulate their metabolic activities across different metabolic pathways [2]. The study of cancer metabolism started with Otto Warburg in the early 20th century who first reported that cancer cells preferentially utilize glycolysis instead of aerobic oxidation for energy even in the presence of sufficient oxygen—a phenomenon known as aerobic glycolysis or the Warburg effect [3]. Since then, researchers have delved into the unique metabolic characteristics of tumors, uncovering their complex and indispensable role in shaping cancer cell fate.

**FIGURE 1:**
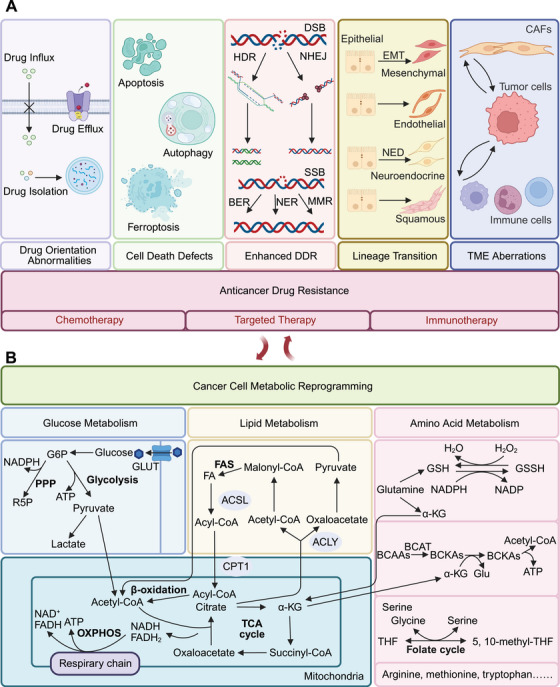
Role of metabolic reprogramming in drug resistance in cancer. Metabolic reprogramming in cancer cells profoundly impacts the responsiveness of these cells to drugs. Cancer cells rewire the flux of the metabolism of the three major nutrients, glucose, lipid, and amino acids, leading to abnormalities in drug orientation, defects in cell death mechanisms, increased DDR, lineage transition, and cross‐talk within the TME. These changes collectively contribute to the development of drug resistance. (A) Metabolic reprogramming in tumors that leads to cancer antitumor drug resistance. Tumor metabolic reprogramming involves alterations in glucose, lipid, and amino acid metabolism. Glycolysis is one of the most classical examples, and reprogramming of the PPP and TCA cycle has also been observed in cancer. Fatty acid synthesis and degradation in tumors are distinct from those in normal tissues. Amino acids such as glutamine, BCAAs, serine, arginine, methionine, and tryptophan also actively participate in tumor metabolic reprogramming. The metabolic products of these three major nutrient pathways—glucose, lipids, and amino acids—can all feed into mitochondrial metabolism, contributing to mitochondrial metabolic reprogramming in cancer. (B) Mechanisms of tumor drug resistance resulting from metabolic reprogramming. The mechanisms that contribute to resistance of chemotherapy, targeted therapy and immunotherapy in cancer include changes in intrinsic tumor characteristics and tumor interactions with the tumor microenvironment (TME). The alternation occurring within tumor cells include drug orientation abnormalities, cell death mechanisms defects, increased DDR, and lineage transition. Metabolic interactions between cancerous elements and noncancerous elements can sustain drug resistance. *Created with BioRender.com*. α‐KG, alpha‐ketoglutarate; 5, 10‐methyl‐THF, 5, 10‐methylene‐tetrahydrofolate; ACLY, ATP citrate lyase; ACSL, acyl‐CoA synthetase long‐chain family member; BCAAs, branched‐chain amino acids; BCAT, branched‐chain aminotransferase; BCKAs, branched‐chain α‐ketoacid; BER, base excision repair; CAFs, cancer‐associated fibroblasts; CPT1, carnitine palmitoyltransferase 1; DDR, DNA damage response; DSB, DNA double‐strand break; EMT, epithelial‐to‐mesenchymal transition; FA, fatty acid; FAO, fatty acid oxidation; FAS, fatty acid synthesis; G6P, glucose 6‐phosphate; Glu, glutamate; GLUT, glucose transporter; GSH, glutathione; GSSH, glutathione disulfide; HDR, homology‐directed repair; MMR, mismatch repair; NADP, nicotinamide adenine dinucleotide phosphate; NADPH, nicotinamide adenine dinucleotide phosphate (reduced form); NED, epithelial‐to‐neuroendocrine differentiation; NER, nucleotide excision repair; NHEJ, nonhomologous end joining; OXPHOS, oxidative phosphorylation; PPP, pentose phosphate pathway; R5P, ribose 5‐phosphate; SSB, DNA single‐strand break; TCA cycle, citric acid cycle; THF, tetrahydrofolic acid; TME, tumor microenvironment.

The current understanding of metabolic reprogramming has extended beyond enhanced aerobic glycolysis. This process involves various metabolic pathways including aerobic oxidation of glucose; the pentose phosphate pathway (PPP); lipid synthesis, degradation, and peroxidation; the metabolism of various amino acids such as glutamine, serine, arginine, tryptophan (Trp), methionine, and branched‐chain amino acids (BCAAs); and mitochondrial metabolism which involves various metabolites (Figure 1B).

Therapeutic resistance to anticancer drugs remains a major challenge in clinical oncology. Intriguingly, metabolic changes in cancer cells are closely associated with the failure of anticancer drug treatments, including chemotherapy, targeted therapy, and immunotherapy. The mechanisms that contribute to such resistance include changes in intrinsic tumor characteristics and tumor interactions with the tumor microenvironment (TME). Therefore, targeting cancer metabolism is proposed to be an important approach for addressing tumor drug resistance (Figure 1A) [4].

In this review, we focus on anticancer drug resistance mechanisms related to metabolic reprogramming both within tumor cells and in the TME and the upstream regulatory alterations that drive them. Additionally, we summarize the current achievements in the combination of small molecule inhibitors targeting cancer metabolism with present treatments to combat drug resistance, aiming to offer valuable insights for overcoming therapeutic challenges in the clinic.

## 2 Metabolic Reprogramming in Cancer

The Warburg effect is one of the most representative metabolic reprogramming mechanisms for cancer and refers to the phenomenon in which tumor cells preferentially utilize glycolysis—a less efficient, anaerobic pathway—to convert glucose into pyruvate and lactate and generate a limited amount of adenosine triphosphate (ATP), even under aerobic conditions. Hexokinase (HK), phosphofructokinase, and pyruvate kinase (PK) are the key enzymes of glycolysis. Pyruvate from glycolysis enters mitochondria to form acetyl‐CoA under the catalysis of pyruvate dehydrogenase (PDH) and participates in the tricarboxylic acid (TCA) cycle. In addition, a portion of glycolytic intermediates can also enter the PPP [5]; this pathway produces raw materials for the synthesis of nucleotides and the reducing agent nicotinamide adenine dinucleotide phosphate (NADPH), which are both important for tumor cells to survive in hostile microenvironmental stresses [6].

Lipid metabolism is another metabolic pathway that plays a more diverse role in tumor fate than it does in normal tissues. Cancer cells preferentially rely on de novo fatty acid synthesis (FAS) to meet lipid demands and can acquire energy through fatty acid oxidation (FAO). FAS starts from acetyl‐CoA conversion to malonyl‐CoA by acetyl‐CoA carboxylase (ACC). Malonyl‐CoA is the reactive raw material for the elongation of fatty acids (FAs) by fatty acid synthase (FASN). FAO is a multistep catabolic process that converts long chain FAs into acetyl‐CoA for the TCA cycle and oxidative phosphorylation (OXPHOS) to produce ATP. FAs are first activated in the cytosol to fatty acyl‐CoA by fatty acyl‐CoA synthetase. To enter mitochondria for FAO, fatty acyl‐CoA is converted to fatty acyl‐carnitine by carnitine palmitoyltransferase 1 (CPT1) on the outer mitochondrial membrane. It is then translocated across the inner membrane by carnitine/acylcarnitine translocase. On the matrix side, CPT2 reconverts acyl‐carnitine to acyl‐CoA, which is subsequently cleaved into acetyl‐CoA for entry into the TCA cycle. In addition, FA peroxidation is critical for processes such as inflammation, ferroptosis, apoptosis, and autophagy [7]. Moreover, alterations in the metabolism of phospholipids, major components of the plasma membrane (PM), profoundly affect the function of the cell membrane, and the resulting metabolic products can influence downstream signaling pathways.

The metabolism of amino acids in tumors differs significantly from that in normal tissues. Glutamine is a nonessential amino acid that provides nitrogen and carbon for biosynthetic reactions. However, for tumor cells, the importance of glutamine is comparable to that of glucose. Glutaminolysis produces α‐ketoglutarate (α‐KG), which can then enter the TCA cycle to generate ATP and provide materials for biosynthesis. Glutamine also contributes to maintaining redox balance by generating glutathione (GSH), which is an antioxidant that scavenges free radicals and detoxifying agents to protect cells from oxidative damage and maintain intracellular redox homeostasis [8]. The serine synthesis pathway (SSP), which provides a carbon source for one‐carbon metabolism, plays a crucial role in de novo nucleotide synthesis and is upregulated in many resistant cancers [9]. In addition, the catabolism of BCAAs including leucine, isoleucine, and valine, by branched‐chain aminotransferase 1 (BCAT1) facilitates reversible transamination between BCAAs and glutamate. BCAAs themselves can also act as sensors to signal the nutritional state of cells [10].

The metabolism of the aforementioned three major nutrients, glucose, lipid, and amino acids, can contribute to the generation of acetyl‐CoA, which then enters the mitochondria to participate in the TCA cycle, which is coupled with cellular energy production via OXPHOS with the help of electron transport chain (ETC). With the expansion of cancer metabolism research, OXPHOS is found to remain dominant in many tumors and tumor cells with enhanced glycolysis still maintain functional OXPHOS and have the ability to switch between OXPHOS and glycolysis to meet their metabolic demands [3].

## 3 Metabolic Reprogramming‐Driven Mechanisms of Drug Resistance

Tumor cells can resist drugs before they reach their intended targets through anomalous drug influx, efflux, and abnormal drug isolation [11, 12]. Once a drug creates a stressful environment, tumor cells start to initiate anti‐cell death signals [13], activate survival pathways [14], repair cellular damage, and even undergo lineage switching for resistance. All of these reactions can be driven by dysregulated metabolic alternations (Table 1).

**TABLE 1:** Mechanisms of cancer drug resistance induced by metabolic reprogramming.

Resistance mechanism		Metabolic drivers	Drugs	References
Dysregulation of drug transport and compartmentalization	Reduced drug uptake	Cholesterol synthesis	TMZ	[15]
		Phospholipid membrane remodeling	DOX	[16]
	Increased drug efflux	Glycolysis	Etoposide, cisplatin	[17, 18]
		GSH biosynthesis	DOX, venetoclax	[19, 20]
	Lysosome compartment	Hypoxia	DOX	[21]
Evasion of cell death programs	Apoptosis dysregulation	FAO	Paclitaxel	[22]
		Mitochondrial metabolism	Cisplatin	[23, 24]
		PPP	Cisplatin, paclitaxel	[24]
		GSH biosynthesis	TKI	[25]
		Glycolysis	EGFR‐TKI	[26, 27]
		TG synthesis	Sorafenib	[28]
	Ferroptosis suppression	FA peroxidation	Cisplatin, oxaliplatin, paclitaxel, 5‐FU, TKI	[29, 30, 31]
		Iron metabolism	Sorafenib, PARPi, DOX	[32, 33, 34]
		Lipid peroxidation degradation	Etoposide, 5‐FU, tamoxifen, TKI, cisplatin, oxaliplatin, TMZ	[35, 36, 37, 38, 39, 40, 41, 42, 43, 44]
	Autophagy induction	GSH synthesis	KRAS inhibitor, sorafenib	[45, 46]
		FAO	Vemurafenib, pazopanib	[47, 48]
		Glycolysis	Bevacizumab, platinum	[49, 50]
		Branched‐chain amino acid metabolism	Cisplatin	[51]
		Glutaminolysis	TMZ	[52]
Enhanced DNA damage tolerance	Improved recognition and initiation of DNA damage	Mitochondrial metabolism	TMZ, PARPi	[53, 54]
		Glycolysis	Niraparib	[55]
	Promotion of repair effects	Glycolysis	Cisplatin, GEM	[56, 57, 58]
		Nucleotide biosynthesis	DOX	[59]
		SSP	5‐FU	[60, 61, 62]
		Glutaminolysis	GEM	[58, 63]
		PPP	GEM, cisplatin	[58, 64]
Cell lineage transition		Glycolysis	Cisplatin	[65]

Cancer drug resistance can be induced by metabolic reprogramming, which affects the efficacy of chemotherapy, targeted therapy, and immunotherapy.

Abbreviations: 5‐FU, 5‐fluorouracil; DOX, doxorubicin; EGFR‐TKI, epidermal growth factor receptor tyrosine kinase inhibitor; FA, fatty acid; FAO, fatty acid oxidation; GEM, gemcitabine; GSH, glutathione; PARPi, poly (ADP‐ribose) polymerase inhibitor; PPP, pentose phosphate pathway; TG, triglyceride; TKI, tyrosine kinase inhibitors; TMZ, temozolomide.

### 3.1 Dysregulation of Drug Transport and Compartmentalization

**FIGURE 2:**
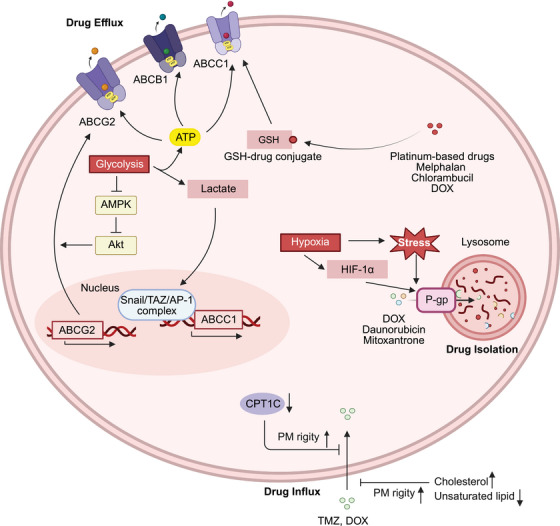
Metabolic mechanisms that influence drug orientation in tumor cells. Abnormal metabolic flux in tumor cells, including aberrant glycolysis as well as subsequent redox imbalance, hypoxia and low pH in the TME, can affect drug transport to their corresponding targets. By increasing drug efflux, decreasing drug influx or isolating drugs in lysosomes, tumor cells can prevent drugs from accumulating to their effective concentrations. *Created with BioRender.com*. AMPK, AMP‐activated protein kinase; AP‐1, activator protein‐1; CPT1C, carnitine palmitoyltransferase 1C; DOX, doxorubicin; GSH, glutathione; HIF‐1α, hypoxia‐inducible factor‐1α; MRP1, MDR‐associated protein 1; P‐gp, P‐glycoprotein; PM, plasma membrane; TAZ, transcriptional coactivator with PDZ‐binding motif; TMZ, temozolomide.

Metabolic reprogramming can affect the influx, efflux and compartmentalization of drugs in cells and prevent drugs from interacting with their targets (Figure 2).

Lipid metabolic reprogramming plays a crucial role in decreasing the uptake of drugs by cells. Temozolomide (TMZ)‐resistant glioblastoma multiforme (GBM) cells rewire lipid metabolism to increase the cholesterol and decrease the unsaturated lipid ratio in the PM, which results in increased rigidity of the PM and reduced drug uptake via passive diffusion or endocytosis [15]. The silencing of CPT1C, an isoform of CPT1, increases PM rigidity, resulting in a reduction in doxorubicin (DOX)‐triggered cell death in breast cancer [16].

Another mechanism that isolates drugs from their intracellular targets is active efflux into the extracellular space, which is mediated primarily by the ATP‐binding cassette (ABC) transporter family of transmembrane proteins [14]. Three proteins in this family, namely, multidrug resistance protein 1 (MDR1; also known as P‐glycoprotein, P‐gp and ABCB1), MDR‐associated protein 1 (MRP1; also known as ABCC1), and breast cancer resistance protein (BCRP; also known as ABCG2), can function as the energy‐dependent exporters of anticancer drugs [66]. In non‐small cell lung cancer (NSCLC) cells, lactate from elevated glycolysis acts as a signaling molecule in the formation of the activated transcription complex at the *ABCC1* promoter, promoting the efflux of etoposide from the cells [17]. Glycolysis has also been shown to promote the transport of ABCG2 to the cell membrane by suppressing AMP‐activated protein kinase (AMPK) and subsequently activating the Akt pathway [18]. Apart from glycolysis, upregulated GSH biosynthesis enhances ABCC1‐mediated drug efflux since ABCC1 prefers to extrude drugs in the presence of GSH and/or in the form of GSH conjugates [19, 67, 68, 69]. Disruption of GSH metabolism in acute myeloid leukemia (AML) impairs ABCC1‐mediated efflux of the BCL2 apoptosis regulator (BCL‐2) inhibitor venetoclax, thereby sensitizing malignant cells to this targeted therapy [20]. In summary, tumor metabolic reprogramming exerts dual regulatory control over ABC transporters, directly activating the transcription of their encoding genes and facilitating their function. In promoting the expression of the ABC transporter family, the function of metabolic reprogramming has been shown to involve signal transduction rather than providing energy or substrates, therefore, further exploration is needed.

Lysosomes are compartments where cell components are degraded and recycled; in addition to their housekeeping function, they can sequester chemotherapeutic agents with weak base properties such as DOX, daunorubicin, and mitoxantrone, thereby decreasing the interactions of anticancer drugs with their intended targets [11, 70]. Mechanistically, P‐gp can be expressed on the lysosomal membrane and transport drugs into lysosomes. Short‐term exposure to tumor stressors, such as hypoxia and nutrient starvation, can redistribute P‐gp to the lysosomal membrane. After longer exposure, hypoxia inducible factor 1 subunit alpha (HIF‐1α) expression induces greater P‐gp accumulation in the lysosomal membrane [21]. These two mechanisms can facilitate the effective sequestration of drugs in lysosomes, resulting in drug resistance.

### 3.2 Evasion of Cell Death Programs

**FIGURE 3:**
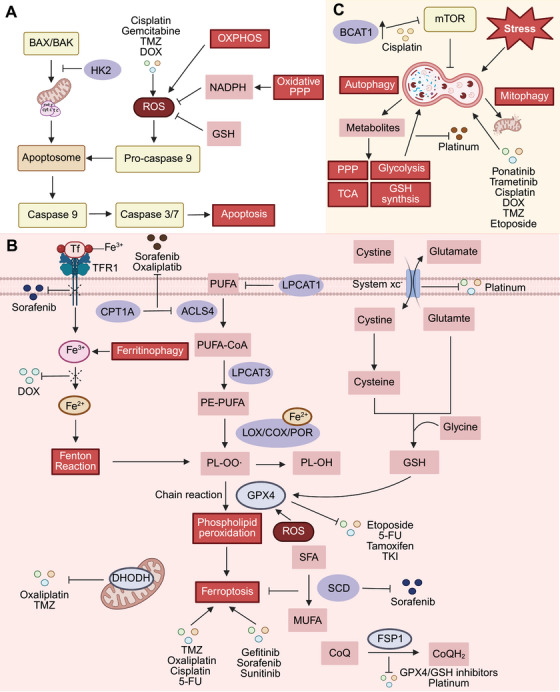
Cell death defects contribute to drug resistance. (A) Tumor cells develop resistance to ROS‐dependent anticancer drugs by undergoing metabolic reprogramming, which includes upregulating glycolysis, downregulating OXPHOS, and increasing BCAA catabolism to suppress ROS production. (B) The metabolism and autophagy processes of tumor cells influence each other, thereby limiting the efficacy of anticancer drugs. (C) The three pivotal components of ferroptosis—iron accumulation, phospholipid peroxidation, and the defense system—all play crucial roles in drug resistance. *Created with BioRender.com*. ACLS4, acyl‐CoA synthetase long‐chain family member 4; BAK, BCL2 killer; BAX, BCL2‐associated X; BCAAs, oxidative phosphorylation; COX, cyclooxygenase; CPT1A, carnitine palmitoyltransferase 1A; DHODH, dihydroorotate dehydrogenase, TMZ, temozolomide; DOX, doxorubicin; FSP1, ferroptosis suppressor protein 1; GPX4, glutathione peroxidase 4; GSH, glutathione; GSH, glutathione; HK2, hexokinase 2; LOX, lipoxygenase; LPCAT1, lysophosphatidylcholine acyltransferase 1; LPCAT3, lysophosphatidylcholine acyltransferase 3; MUFAs, monosaturated fatty acids; NADPH, nicotinamide adenine dinucleotide phosphate; OXPHOS, oxidative phosphorylation; PE‐PUFAs, phosphatidylethanolamines; PL‐OO·, phospholipid peroxyl radical; POR, cytochrome P450 oxidoreductase; PPP, pentose phosphate pathway; PUFA, polyunsaturated fatty acids; ROS, reactive oxygen species; SCD, stearoyl‐CoA desaturase; SFA, saturated fatty acids; Tf, transferrin; TFR1, transferrin receptor 1.

One of the primary causes of drug resistance in cancer cells is their ability to prevent cell death. Programmed cell death (PCD) is the regulated death of a cell through a series of signaling molecules. Apoptosis, which is one of the most classic and prevalent types of PCD, is the end point of various resistance pathways. Ferroptosis is a metabolism‐dependent form of PCD. The occurrence of both types of PCD requires the participation of reactive oxygen species (ROS) and the normal regulation of the intracellular redox state. In addition, the aforementioned two mechanisms, autophagy is a survival mechanism that is employed by cancer cells in hostile microenvironments. All of these three types of PCD link metabolism to drug resistance (Figure 3).

#### 3.2.1 Apoptosis Dysregulation

Apoptosis is executed by a group of cysteine dependent, aspartate‐directed proteases known as caspases. Various chemotherapeutic and targeted therapeutic agents exploit ROS to induce apoptosis in tumor cells [71, 72]. These ROS‐related anticancer agents increase the permeability of the mitochondrial membrane, facilitating the release of proapoptotic factors, such as cytochrome (cyt) *c* and procaspase 9, to activate effector caspases [73, 74, 75]. However, recent study revealed that increased acetyl‐CoA from FAO acetylates and activates the transcription factor signal transducer and activator of transcription 3 (STAT3). STAT3 upregulates the expression of acyl‐CoA synthetase long‐chain family member 4 (ACSL4), leading to increased phospholipid synthesis in mitochondrial membranes and increased mitochondrial integrity in the context of chemotherapy‐induced apoptosis [22]. Additionally, reliance on glycolysis instead of OXPHOS in tumors leads to decreased ROS production. In addition, aberrant states of ROS sensors in tumor cells can disrupt mitochondrial translation and impair activity, leading to reduced homeostatic mitochondrial and cellular ROS levels [23]. Metabolic reprogramming can also initiate a range of antioxidant defense mechanisms to resist ROS‐mediated apoptosis. NADPH from the activated PPP leads to ROS clearance and multidrug resistance [24]. The upregulation of BCAT1 facilitates glutamate–cysteine ligase catalytic subunit‐mediated GSH synthesis and leads to tyrosine kinase inhibitor (TKI) resistance in human epidermal growth factor receptor (EGFR) mutant lung cancer cells [25]. Therefore, thoroughly investigating any metabolic reprogramming that affects ROS levels is essential for addressing drug resistance.

In addition to indirectly influencing drug resistance by modulating ROS levels, metabolic enzymes or metabolic products altered in metabolic reprogramming have been shown to directly interfere with apoptosis. For example, hexokinase 2 (HK2), the isoform of HK, can prevent the binding of the proapoptotic protein Bax to mitochondria, thereby inhibiting the release of cyt *c* and leading to erlotinib resistance in NSCLC [26, 27]. Similarly, de novo triglyceride (TG) synthesis driven by glycerol‐3‐phosphate acyltransferase 3 can stimulate the NF‐κB/BCL‐2 antiapoptotic signaling pathway, leading to the resistance of hepatocellular carcinoma (HCC) cells to sorafenib treatment [28].

#### 3.2.2 Ferroptosis Suppression

Ferroptosis is an iron‐dependent form of PCD that is characterized by lethal lipid peroxidation [76]. In‐depth investigations into ferroptosis have revealed its association with the pharmacological effects of multiple chemotherapeutic agents, targeted drugs, and immunotherapies [77, 78]. Hence, attenuation of ferroptosis in cancer cells has been identified as a key factor that contributes to the development of drug resistance. Metabolic reprogramming significantly influences this suppression of ferroptosis through disruptions in lipid metabolism, iron regulation and defense mechanisms.

The primary substrates of lipid peroxidation during ferroptosis are polyunsaturated fatty acids (PUFAs), which are abundant in cell membranes [79]. ACSL4 catalyzes the conversion of PUFAs into PUFA‐CoA. CPT1A can downregulate ACSL4 expression and the downregulation of ACSL4 has been found to mediate ferroptosis‐related resistance to sorafenib in HCC or oxaliplatin in colorectal cancer (CRC) [29, 80, 81]. PUFA‐CoA intermediates are subsequently catalyzed into PUFA‐phosphatidylethanolamines (PE‐PUFAs) by lysophosphatidylcholine acyltransferase 3 (LPCAT3) [82]. PE‐PUFAs in phospholipids can then be dioxygenated into phospholipid peroxyl radicals (PL‐OO·) through either the Fenton reaction (the generation of both hydroxyl radicals and higher oxidation states of iron) [83] or by enzymes such as lipoxygenases (LOXs) [7], cyclooxygenases (COXs) [84], and/or cytochrome P450 oxidoreductase (POR) [76]. These newly formed free radicals promote further phospholipid peroxidation, initiating a chain reaction that compromises the membrane structure and ultimately leads to cell death [79]. Since only PUFAs can undergo peroxidation, drug‐resistant tumor cells dynamically remodel membrane phospholipids. For example, lysophosphatidylcholine acyltransferase 1 (LPCAT1) can incorporate more saturated fatty acids (SFAs) into the membrane and reduce PUFA levels in the membrane [85]. The activity of stearoyl‐CoA desaturase (SCD), which is the rate‐limiting enzyme in the production of monounsaturated fatty acids (MUFAs), can also help prevent ferroptosis [86, 87]. High expression of SCD has been observed in multiple cancers, such as gastric cancer [30], breast cancer [88], NSCLC [89] and HCC [90], in response to chemotherapy [30, 88, 89] and targeted therapy [88, 90]. Furthermore, in antimitotic drug‐resistant cancer cells, PUFAs can be sequestered into lipid droplets, preventing their incorporation into phospholipids and thereby inhibiting ferroptosis [31].

Iron has a crucial impact in various stages throughout the whole process: (1) facilitating the nonenzymatic Fenton reaction pathway, (2) acting as a catalyst in enzymatic reactions driven by LOXs or POR, and (3) contributing to ROS generation. Abnormalities in iron carrier transferrin recycling and membrane localization have been found to contribute to sorafenib resistance in HCC [32]. Another pathway for cellular iron uptake, ferritinophagy, which refers to the autophagic degradation of the intracellular iron storage protein ferritin, can alter intracellular labile iron levels, thereby mediating ferroptosis‐related resistance in tumor cells [33]. Since only Fe^2^⁺, rather than Fe^3^⁺, can participate in lipid peroxidation, the inhibition of Fe^3^⁺‐to‐Fe^2^⁺ conversion has been found to promote DOX resistance in breast cancer [34].

Cells currently employ four key mechanisms to protect against ferroptosis: (1) the GSH peroxidase 4 (GPX4)–GSH system in the cytoplasm and mitochondria [91]; (2) the ferroptosis suppressor protein 1 (FSP1; also known as AIF family member 2–ubiquinol (CoQH_2_) system in the PM [92, 93]; (3) the dihydroorotate dehydrogenase (DHODH)–CoH_2_ system in mitochondria [94]; and (4) the GTP cyclohydrolase 1 (GCH1)–tetrahydrobiopterin (BH_4_) system [95, 96]. These pathways effectively trap lipid peroxyl radicals, thereby halting the propagation of the chain reaction [91, 92, 93, 94, 95, 96]. GPX4 is the enzyme that transforms the peroxo bond in lipid peroxides into a hydroxyl group. In the classical regulation of ferroptosis, cysteine is imported into cells via the cysteine/glutamate antiporter (system Xc^−^ or SLC7A11). Within cells, cysteine supports the synthesis of GSH, which acts as a cofactor for GPX4, reducing PL‐OOHs to their corresponding alcohols (PL‐OHs) to mitigate cellular damage [76]. ROS from OXPHOS can activate the p38/ serum‐ and glucocorticoid‐inducible kinase 1 (SGK1) axis, which in turn reduces the interaction between GPX4 and its E3 ubiquitin ligase, thereby stabilizing GPX4 expression [35]. Dysregulation of GPX4 expression can lead to resistance to multiple therapeutic strategies, such as etoposide in NSCLC [35], 5‐fluorouracil (5‐FU) in gastric cancer [36, 37], tamoxifen in breast cancer [97] and TKIs in leukemia [38]. Moreover, the overexpression or abnormal membrane localization of SLC7A11 has been found to mediate chemoresistance to platinum‐based drugs [39, 40, 41]. CoQH_2_, which captures lipid peroxyl radicals, is regenerated from CoQ by the NAD(P)H‐dependent oxidoreductase FSP1 [92, 93]. The stability of FSP1 has been found to contribute to platinum resistance in ovarian cancer [42]. Increased expression of FSP1 enhances the resistance of acute lymphoblastic leukemia cells to agents that target the GSH‐dependent antiferroptosis pathway, allowing these cells to evade ferroptosis through an alternative mechanism [98]. DHODH is an additional enzyme that is capable of reducing CoQ to CoQH_2_ in mitochondria. Chemotherapy‐mediated activation of EGFR signaling can stabilize DHODH in HCC via the lysyl oxidase‐like 3/translocase of the outer membrane 20/adenylate kinase 2 pathway [43]. Targeting the proline‐rich 11–DHODH axis can enhance lipid peroxidation and alter DHODH‐mediated mitochondrial morphology, thereby promoting ferroptosis and increasing TMZ chemotherapy sensitivity in GBM [44]. However, as a new defense mechanism, few studies have reported the relationship between GCH1–BH_4_ and drug resistance, but this could be a promising research direction since GCH1 can produce not only BH_4_ but also CoQH_2_, acting as a robust supplement to the GPX4–GSH system. The induction of ferroptosis is increasingly viewed as a promising strategy for combatting drug resistance since multiple resistant cancers exhibit metabolic vulnerabilities that depend on the ferroptosis suppression mechanisms [99]. However, resistance to various ferroptosis inducers is also emerging, highlighting the need for additional combination therapies to overcome the drug resistance of cancer cells [100, 101].

#### 3.2.3 Autophagy Induction

Autophagy is a self‐degradative process that eliminates and recycles cytoplasmic waste as well as damaged or redundant proteins and organelles, generating new energy and materials that are vital for cell survival [102]. Through selective degradation under specific conditions, autophagy is helpful for counteracting cytotoxic stresses, such as nutrient starvation, hypoxia, oxidative stress, and infection [103]. Unlike normal cells that undergo apoptosis under stressful conditions, tumor cells prefer to induce autophagy to maintain intracellular homeostasis and survive in adverse environments [104]. Studies have revealed that targeted therapy (e.g., ponatinib [105] and trametinib [97]) and chemotherapy (e.g., cisplatin, DOX, TMZ, and etoposide) [106] can induce autophagy. On the one hand, enhanced autophagy can directly generate the substrates required for metabolic reprogramming [107, 108]. For example, the glutamate, cysteine, and glycine produced by autophagy can be utilized for GSH synthesis [45, 46]. On the other hand, autophagy helps maintain the metabolic state required for the highly active physiological status of tumor cells. Mitophagy serves as a prototypical example. Thyroid cancer cells that rely on FAO for energy require mitophagy to maintain proper mitochondrial respiration [47]. Mitophagy can also restrain excessive oxidative reactions to prevent excessive accumulation of ROS [48]. Intriguingly, these tumor‐associated metabolic reprogramming events can, in turn, promote the induction of autophagy. For example, lactate from elevated glycolysis in CRC cells facilitates autophagosome maturation via histone lactylation, ultimately leading to resistance to bevacizumab therapy [49]. The inhibition of acetate‐dependent acetyl‐CoA synthetase 2 (ACSS2), which reduces acetate metabolism and suppresses glycolysis, significantly activates autophagy in ovarian cancer (OC) cells and exerts antitumor effects on platinum [50]. In addition to regulating autophagy through metabolic byproducts, cells can modulate autophagic activity through sensing their intracellular energy status via the mammalian target of rapamycin (mTOR) signaling [109]. Under low‐energy conditions, such as those experienced by cancer stem cells within the hypoxic niche, AMPK is activated, leading to mTOR inactivation and subsequent autophagy induction [109, 110]. In cisplatin‐resistant HCC, BCAT1 downregulates mTOR, thus promoting autophagy [51].

Nevertheless, given that its overactivation also leads to PCD or the degradation of critical factors sustaining tumor maintenance, autophagy can significantly increase the efficacy of chemotherapy. Glutamine deprivation in highly glutamine‐dependent GBM stem cells activates autophagy to suppress CD133 expression, thereby disrupting its stemness and reducing the drug resistance of tumors [52]. Taken together, these findings suggest that the interactions among autophagy, metabolic reprogramming and drug resistance in tumor cells are complicated. On the one hand, while cancer cells depend on autophagy for metabolic reprogramming, they also undergo metabolic changes that initiate autophagy. On the other hand, autophagy plays dual roles in the tumor response to stress: it not only aids tumor survival under adverse conditions but also contributes to cell death. This dual function of autophagy complicates its impact on drug resistance, making it both a facilitator and a mitigator.

### 3.3 Enhanced DNA Damage Tolerance

Multiple antitumor drugs, such as alkylating agents, directly damage DNA through DNA single‐strand breaks (SSBs) or DNA double‐strand breaks (DSBs) [14, 111]. Cells respond to these damages either by undergoing cell death or initiating the DNA damage response (DDR), depending on the extent of the damage. Hence, the efficiency of the DDR in cancer cells significantly affects the efficacy of these drugs. SSBs are repaired through various pathways including base excision repair (BER), nucleotide excision repair (NER), and mismatch repair (MMR), whereas nonhomologous end joining (NHEJ) and homology‐directed repair (HDR) are the mechanisms for repairing DSBs [112, 113]. Cellular metabolism critically influences these processes, from the perception and recognition of feedback signals from damage sites to the recruitment of repair factors to damage sites to the repair of broken DNA strand, critically impacting the cellular response to cancer treatments.

#### 3.3.1 Improved Recognition and Initiation of DNA Damage

After DNA damage occurs, a series of DNA damage sensors can recognize DNA breaks and participate in the early recruitment of repair factors to facilitate the DDR. Poly‐ADP‐ribose polymerase 1 (PARP1) is one of the key early sensors that can bind DDR‐related and DNA metabolism‐related proteins via the polymerization of ADP‐ribose (PAR) units, which results in their recruitment to sites of DNA damage [114]. FAO plays a vital role in this process by providing acetyl‐CoA to facilitate PARP1 acetylation and activation [115]. In addition, PARP1 uses nicotinamide adenine dinucleotide (NAD) as a co‐factor to catalyze PAR units [114]. Cytochrome P450 family 3 subfamily A member 5 (CYP3A5) functions to convert NADH to NAD. Its overexpression can increase the activity of PARP, conferring resistance to TMZ in GBM [53]. The absence of the mitochondrial NAD transporter solute carrier family 25 member 51 (SLC25A51) results in the accumulation of NAD in the nucleus and increased PARP1‐mediated nuclear ADP‐ribosylation [54]. RAD23A is another factor involved in the recognition of DNA damage, and it functions primarily in the NER pathway. Lactate derived from glycolysis can promote the lactylation of histone H4K12 lysine residues, which in turn activates the transcription factor MYC to upregulate RAD23A expression. This enhances the DNA damage repair capacity and contributes to chemoresistance in OC cells [55]. Chromatin relaxation is also a prerequisite for the access of the DDR machinery. In the process of DDR, PDH 1α (PDHE1α) is quickly recruited to chromatin in a PAR‐dependent manner, which drives acetyl‐CoA generation to support local chromatin acetylation around DSBs and increase the formation of relaxed chromatin [116]. However, recent studies have shown that the cancer metabolites 2‐hydroxyglutarate (2‐HG), succinate and fumarate suppress the HDR pathway by masking signals for the proper execution of HDR and the consequent recruitment of key proximal HDR factors [117, 118].

#### 3.3.2 Promotion of Repair Effects

After the formation and recognition of DNA damage, in HDR, the 5′ ends of DSBs are excised to form 3‐terminal single‐stranded DNA under the action of specific nuclease complexes, one of which is the MRE11–RAD50–NBS1 (MRN) complex. Recent studies revealed that lactate from the Warburg effect or glutamine‐derived malate [119] can lactylate MRE11 and NBS1, promoting the formation of the MRN complex and the binding of the DNA strand. The inhibition of MRE 11 or NBS1 lactylation sensitized cancer cells to cisplatin [56, 57].

The final procedure of DDR is break‐induced replication. Although the number of deoxynucleotide triphosphates required for DDR is minimal, their concentration is crucial during the repair process. For example, chemoresistant ovarian cancers exhibit higher purine abundance [59]. Therefore, reprogramming nucleotide biosynthesis in cancer cells plays a key role in the DDR and resistance to genotoxic drugs [120, 121]. Tumor cells predominantly utilize the de novo pathway of nucleotide synthesis to satisfy their extensive metabolic needs for proliferation [112]. De novo synthesis includes a series of enzymatic reactions that utilize ribose‐5‐phosphate, amino acids, one‐carbon units, and CO_2_ as substrates. An increase in endogenous serine synthesis by SSP or exogenous serine uptake leads to 5‐FU resistance in CRC cells [60]. In addition, exogenous serine deprivation can promote the transcription of the serine metabolic enzymes phosphoglycerate dehydrogenase (PHGDH), phosphoserine aminotransferase 1, and phosphoserine phosphatase, which in turn support serine biosynthesis and contribute to 5‐FU resistance [61]. Further study revealed that the COP9 signalosome subunit increases the expression of PHGDH in 5‐FU‐resistant CRC [62]. Glutamine is also integral to the de novo pathway [122]. Increased uptake or synthesis of glutamate contributes to gemcitabine (GEM) resistance in pancreatic cancer [63]. The PPP is another contributor to nucleotide metabolism [58, 64]. A study revealed that DNA damage triggers the activation of ataxia‐telangiectasia mutated (ATM), which is a crucial DDR protein that induces the catalytic function of glucose‐6‐phosphate dehydrogenase (G6PDH), the rate‐limiting enzyme of the PPP, and promotes nucleotide production for DSB repair [123]. Correspondingly, the combined inhibition of the ATM/G6PDH and FMS‐like tyrosine kinase 3 pathways, the latter of which is an activating mutation in AML, results in synthetic lethality [124].

A recent study expanded the role of nucleotide metabolism alterations from merely providing substrates for the DDR to also acting as regulatory molecules in NHEJ [125]. However, nonmaterial‐related functions have been mentioned only in purine nucleotides, and whether pyrimidine nucleotides have similar functions remains to be investigated. Moreover, the epigenetic modification functions of metabolites in the DDR in drug‐resistant tumor cells requires further research. For example, how these products achieve effective concentrations within the nucleus, whether these modifications have inhibitory effects in addition to promoting DDR, and other metabolite modification methods remain to be explored.

### 3.4 Cell Lineage Transition

During the evolution of tumors, cancer cells can acquire new differential patterns via dedifferentiation and transdifferentiation, which are identified as lineage plasticity or phenotypic plasticity. Variations in phenotype include: epithelial‐to‐squamous transition, epithelial‐to‐endothelial transformation, epithelial‐to‐neuroendocrine differentiation, and epithelial‐to‐mesenchymal transition. Lineage plasticity endows tumor cells with the ability to adapt to and survive in the harsh environment created by anticancer treatments, which has been observed in prostate and lung cancers [126, 127]. Since metabolites provide essential building blocks for tumor evolution and actively participate in epigenetic modifications, they play a prominent role in promoting lineage plasticity. For example, a subpopulation of cisplatin‐resistant bladder cancer cells is glycolytic dependent, with lactate production driving squamous differentiation through H3K18 lactylation [65]. Although the relationships among lineage plasticity, metabolism, and drug resistance are well recognized individually, few studies have directly connected all three, leaving much to be explored.

## 4 TME as a Metabolic Hub for Resistance

**FIGURE 4:**
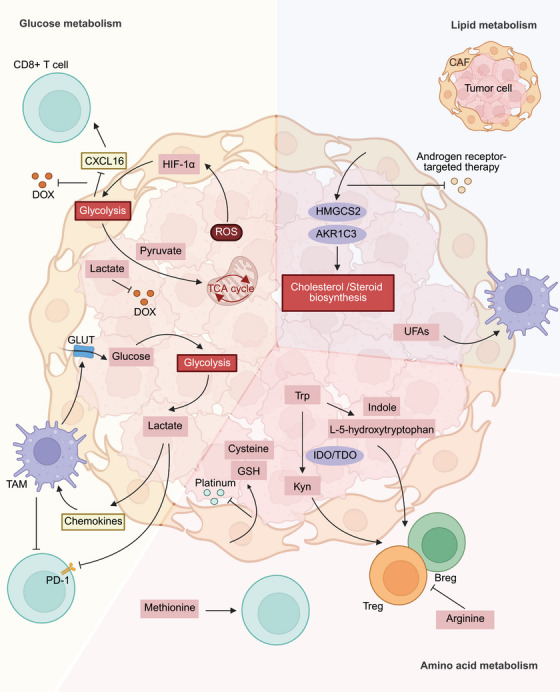
Metabolic crosstalk in the TME facilitates drug resistance. The cellular components of the TME, including CAFs and immune cells, interact with tumor cells through metabolites and metabolic pathways. All three major nutrients, glucose, lipids, and amino acids, participate in these processes. *Created with BioRender.com*. AKR1C3, aldo‐keto reductase family 1 member C3;, UFA, unsaturated fatty acids; CAFs, cancer‐associated fibroblasts; DOX, doxorubicin; GLUT, glucose transporter; GSH, glutathione; HIF‐1α, hypoxia‐inducible factor‐1α; HMGCS2, 3‐hydroxy‐3‐methylglutaryl‐coenzyme A synthase 2; IDO/TDO, indoleamine 2,3‐dioxygenase 1 and tryptophan 2,3‐dioxygenase 2; Kyn, kynurenine; ROS, reactive oxygen species; TAM, tumor‐associated macrophage; TCA cycle, citric acid cycle; Trp, tryptophan.

It is widely accepted that tumor tissues are complex entities that include not only cancerous elements but also noncancerous elements, all of which form a microenvironment that supports disease progression. Cancer cells engage in metabolic interactions with other cells, notably cancer‐associated fibroblasts (CAFs) and immune cells, to sustain drug resistance (Figure 4 and Table 2).

**TABLE 2:** Drug resistance related metabolic crosstalk within TME.

Metabolic crosstalk within TME	Metabolic drivers	Drugs	References
Metabolic crosstalk with CAFs	Glycolysis	Oxaliplatin, DOX	[128, 129]
	Ferroptosis	DOX	[130]
	Cholesterol biosynthesis	Enzalutamide	[131]
Immunosuppressive metabolic crosstalk	Glycolysis	ICB	[132, 133]
	FAO	ICB	[134]
	Glutaminolysis	ICB	[135]
	Tryptophan metabolism	ICB	[136]
	Methionine metabolism	ICB	[137]
	Arginine depletion	ICB	[138, 139, 140, 141]

Cancer cells interact metabolically with CAFs and immune cells in the TME to promote drug resistance.

Abbreviations: CAFs, cancer‐associated fibroblasts; DOX, doxorubicin; FAO, fatty acid oxidation; ICB, immune checkpoint blockade; TME, tumor microenvironment.

### 4.1 Metabolic Crosstalk with CAFs in the TME

CAFs are activated fibroblasts in the TME that transform from normal fibroblasts via a mechanism that is regulated by growth factors, cytokines and other signaling molecules produced by cancer cells [142, 143, 144]. This process requires support from the metabolic reprogramming of CAFs, which provides various nutrients. For example, a recent study has shown that CAFs rely on NAD kinase 2 (NADK2)‐mediated mitophagy to supply proline for their activation [145]. Tumor cell‐derived ROS stabilize HIF‐1α and activate NF‐κB to increase glycolysis in CAFs, which is referred to as a “pseudohypoxic” condition for CAFs [143]. Glycolytic CAFs generate metabolites, such as pyruvate, ketone bodies, lactates, and glutamates, which are subsequently utilized by adjacent tumor cells [146, 147]. Some tumor cells can take up these metabolites for the TCA cycle, and this phenomenon is referred to as the “reverse Warburg effect” [148, 149]. Notably, this enhanced mitochondrial metabolism can induce drug resistance through the promotion of cancer stem cell survival or the inhibition of cancer cell apoptosis [150, 151]. Metabolites derived from CAFs function not only as nutrients for tumor cells, but also as regulatory factors to modulate tumor resistance. For example, lactate derived from CAFs acts, on the one hand, as an autocrine regulatory factor to promote IL‐8 secretion, thereby enhancing DNA damage repair in pancreatic cancer cells [128]. On the other hand, it can be taken up by tumor cells and contribute to histone lactylation, ultimately suppressing chemotherapy‐induced PCD [130]. Glycolytic CAFs can also influence immune cell function, for example, by upregulating CXCL16, a T cell‐retention chemokine that restricts CD8⁺ T cells to the tumor periphery, and suppressing DOX‐induced immunogenic cell death [129, 152]. In addition to regulating glucose metabolism, in prostate cancer, CAFs significantly increase cholesterol and steroid biosynthesis pathways by upregulating the expression of the key enzymes 3‐hydroxy‐3‐methylglutaryl‐coenzyme A synthase 2 (*HMGCS2*) and aldo‐keto reductase family 1 member C3 (*AKR1C3*), thus contributing to resistance to androgen receptor‐targeted therapy [131]. Furthermore, CAFs can release GSH and cysteine into the TME; these substances are taken up by NSCLC and OC cells to counteract the effects of platinum‐based therapies [153, 154, 155]. Beyond metabolic products, a hallmark function of CAFs is the secretion of exosomes, which for example deliver microRNAs (miRNAs) that epigenetically regulate tumor cell metabolism, such as those that modulate ferroptosis‐related pathways and ultimately contribute to chemotherapy resistance [156, 157]. Collectively, these findings indicate that metabolic crosstalk among CAFs, tumor cells and immune cells triggered by metabolic reprogramming in the TME has an indispensable effect on drug resistance.

### 4.2 Immunosuppressive Metabolic Crosstalk in the TME

Immunosuppressive metabolic reprogramming within the TME is considerably complex and can contribute to immunotherapy resistance. It involves multiple metabolic pathways, including those of glucose, lipid, and amino acid metabolism and is driven by various components of the TME, involving interactions between tumor cells and different immune cell subsets.

Glycolysis represents a pivotal metabolic pathway that suppresses antitumor immune responses. First, lactate derived from tumor cell glycolysis promotes themselves secreting chemokines and facilitating the recruitment of tumor‐associated macrophages (TAMs) [158]. The recruited TAMs are further polarized under the influence of tumor‐derived lactate [159], leading to impaired infiltration of CD8⁺ T cells and reduced interferon‐γ (IFN‐γ) production [132]. Lactate can also directly suppress PD‐1 expression on CD8⁺ T cells [133], thereby attenuating immune cytotoxic function. TAMs can act as the facilitators of tumor glycolysis by enhancing glucose uptake and glycolysis‐related enzymes activity in tumor cells [160, 161]. Activated T cells also rely on elevated glycolysis to support their functions. Glycolysis supports IFN‐γ production in T cells and enhances tumor sensitivity to immune checkpoint blockade (ICB) [162]. Therefore, to exert potent antitumor effects, these cytotoxic immune cells need to persistently compete with both tumor cells and other immunosuppressive immune cells for metabolic substrates [138]. However, recent studies have shown that redirecting T cell metabolism toward OXPHOS during the effector phase [163], or toward the PPP during the differentiation phase [164], can improve the efficacy of immunotherapy. Given that T cell differentiation is a tightly regulated and stage‐specific process [165], further investigation is required to determine the optimal timing and metabolic direction of such interventions to maximize therapeutic benefit.

In contrast to tumor cells and antitumoral M1 TAMs that rely on glycolysis, protumoral M2 TAMs typically exhibit elevated levels of OXPHOS [166]. However, a recent study revealed that, under CD40 signaling, acetyl‐CoA generated from FAO which is normally associated with M2 polarization facilitates the acetylation of proinflammatory and antitumor–associated genes. The maintenance of FAO is dependent on CD40‐induced glutamine‐to‐lactate conversion and the fine‐tuning of NAD+/NADH ratio [166]. Tumor cells can act as sources of long‐chain unsaturated fatty acids (UFAs) for fatty‐acid‐binding protein 5 (FABP5)‐dependent TAMs [167]. In addition to TAMs, cytotoxic CD8⁺ T cells have been shown to rely on FABP5 to uptake efficient FAs for FAO, thereby fueling mitochondrial respiration and supporting antitumor functions [168]. However, in tumor cells, FAO is exploited to resist immune attack. In response to cytotoxic stress, tumor cells upregulate CPT1A, leading to enhanced FAO and the activation of prosurvival signaling [134]. In contrast to FAO, enhanced lipid synthesis in CD8⁺ T cells leads to abnormal lipid accumulation and increased lipid droplet formation, thereby promoting their senescence and functional exhaustion [169].Both tumor cell–derived PD‐1 and ACC within the TME have been implicated in promoting these metabolic changes [170, 171].

The reprogramming of amino acid metabolism within the TME is also a critical factor for the efficacy of cancer immunotherapy. In this process, the metabolism of glutamine, Trp, methionine, and arginine serve as the key regulators. As a critical energy source within the TME, glutamine is not only heavily reliant upon by tumor cells but is also essential for the activation and proliferation of immune cells during immune responses. This dynamic tug‐of‐war between cancer and immune cells affects the efficacy of immune‐related cancer therapies [135]. Trp is metabolized primarily through three major pathways in the body. The indoleamine 2,3‐dioxygenase 1 and Trp 2,3‐dioxygenase 2 (IDO/TDO)‐mediated kynurenine (Kyn) pathway constitutes the major route of Trp metabolism. The metabolite Kyn functions as a ligand to activate the transcription factor aryl hydrocarbon receptor (AHR) to drive the generation of Tregs, Bregs, immunosuppressive myeloid cells, and PD‐1 upregulation in CD8+T cells, which is further enhanced under Trp‐depleted conditions [136, 172, 173, 174]. Therefore, the use of IDO inhibitors can downregulate PD‐1 expression and activate CD8⁺ T cells by restoring Trp levels [174]. However, a recent study has shown that IDO inhibition can paradoxically protect tumor cells from the detrimental effects of T cell‐derived IFN‐γ, thereby contributing to the failure of IDO inhibitor‐based therapies [175]. Accordingly, a deeper understanding of this pathway is essential for developing effective strategies to circumvent ICB resistance. In addition to Kyn, Trp‐derived L‐5‐hydroxytryptophan and indoles can also stimulate AHR activation and exert immunosuppressive functions similar to those of Kyn [176, 177, 178]. Methionine can be converted into S‐adenosylmethionine, a universal methyl donor, through the catalysis of methionine adenosyltransferase 2A. However, the ability of methionine intervention to overcome ICB resistance appears to be subject to strict contextual limitations. In the “cold” TME of a subgroup of osteosarcoma (OS) patients with methylthioadenosine phosphorylase deletion, methionine restriction triggers PD‐L1 expression in OS cells and activates the immune‐related signaling pathways to attract CD8+ T cells [137]. However, some other tumor cells outcompete T cells for methionine, thereby compromising T cell‐mediated immunity. Methionine supplementation has been shown to restore T cell function and enhance antitumor immune responses [179]. Arginine depletion has been found primarily to activate the immunosuppressive functions of TAMs, Tregs, and tumor‐associated neutrophils, thereby compromising the efficacy of immunotherapy [138, 139, 140, 141]. Therefore, the role of amino acid metabolism in cancer immunotherapy should be studied in a more amino acid‐ and tumor‐specific manner [180].

## 5 Key Drivers of Metabolic Reprogramming in Resistance

To effectively overcome drug resistance caused by metabolic reprogramming, understanding not only the drug resistance mechanisms related to metabolism but also the upstream factors that drive metabolic reprogramming is essential. Among the key drivers, genetic and transcriptional alterations have been implicated among the key drivers.

### 5.1 Aberrant Genetic Alterations

Classical oncogene and tumor suppressor gene aberrations have been identified as drivers of metabolism‐mediated drug resistance. The oncogene *MYC* functions as a transcription factor that regulates metabolic adaptation. MYC upregulates key glycolytic enzymes, thereby contributing to chemoresistance in various cancers [181, 182]. In glutamine‐addicted tumors, MYC promotes the expression of the key enzyme glutaminase 1 (GLS1) in glutaminolysis, sustaining the high metabolic activity as well as antioxidant capacity to fight against ferroptosis [81, 183]. Similarly, activated KRAS in CRC cells induces glutamine dependency for survival, accompanied by enhanced glutaminolysis. This metabolic shift suppresses demethylases and leads to epigenetic alterations, increasing stemness and reducing sensitivity to 5‐FU [184]. The downregulation of the tumor suppressor gene, p53, for example, represents another genetic alteration that leads to resistance‐promoting metabolic changes. In glioma, decreased p53 expression enhances glycolysis and TMZ resistance [185].

Mutations in metabolism‐related genes can also drive metabolic alterations in tumors and drug resistance. Succinate dehydrogenase (SDH), fumarate hydratase (FH), and isocitrate dehydrogenase (IDH) are enzymes that are frequently mutated in various cancers. SDH deficiency leads to the accumulation of succinate and disrupts the TCA cycle. The accumulation of succinate can promote the accumulation of oncogenic proteins and lead to resistance in AML [186]. FH mutations result in fumarate accumulation which inhibits PTEN to activate PI3K/AKT signaling and sensitivity to sunitinib in renal cell carcinoma [187]. Mutations in IDH lead to the production of 2‐HG and often lead to ICB resistance in cholangiocarcinoma [188]. Fortunately, tumors with these specific genetic alterations exhibit the specific metabolic dependencies, therefore creating unique vulnerabilities and therapeutic opportunities.

Aberrations in oncogenes and tumor suppressor genes fundamentally shape the metabolic phenotype of cancer cells. In addition, alterations in metabolism‐related genes can lead to distinct metabolic states in tumors, contributing to therapy resistance. However, studies directly linking metabolic reprogramming, genetic alterations, and drug resistance remain limited and warrant further investigation.

### 5.2 Aberrant Epigenetic Patterns

Epigenetic regulators can modulate the transcription of metabolic enzymes or signaling proteins. Four classic epigenetic mechanisms, DNA methylation, histone modifications, chromatin remodeling, and noncoding RNAs (ncRNAs) contribute to resistance–associated metabolic rewiring.

DNA methylation involves the addition of a methyl group to the 5‐carbon of cytosine residues within CpG dinucleotides and typically leads to gene silencing. Promoter hypermethylation in HCC leads to low expression of oxoglutarate dehydrogenase‐like, a rate‐limiting component of the mitochondrial OGDH complex. An increased α‐KG to citrate ratio from this TCA cycle aberration promotes de novo lipogenesis and enhances NADPH and GSH production to support the cellular antioxidant defense system [189].

Histones, the structural proteins of chromatin, undergo various posttranslational modifications, including acetylation, methylation, phosphorylation, and ubiquitination to influence gene expression. The nicotinamide N‐methyltransferase‐mediated reduction in H3K27me3 epigenetically activates aldehyde dehydrogenase 3A1 (ALDH3A1), leading to increased lactate levels and contributing to resistance to EGFR‐TKIs in NSCLC [190]. EGFR‐TKI resistance is also linked to the derepression of glycolysis‐related genes, which is mediated by α‐KG‐dependent demethylation of H3K27 [191]. In human epidermal growth factor receptor 2 (HER2) + breast cancer, H3K27me3 and H3K4me3 modifications regulate the transcriptional activity of key genes involved in lipid metabolism, contributing to trastuzumab resistance [192]. Another form of histone modification, acetylation, promotes an open chromatin state and increases chromatin accessibility for gene expression. CBP/P300 acetylates histone H3K27 to increase the transcription of CPT1A, thereby increasing FAO and mitigating the cytotoxic effects of tamoxifen in estrogen receptor (ER) + breast cancer cells [193]. Histone deacetylases, including sirtuin 6 (SIRT6) and histone deacetylase 11 (HDAC11), can promote glycolysis by suppressing the expression of intermediate signaling molecules, which in turn contributes to targeted therapy resistance in cancer cells [26, 194].

The chromatin remodeling factor, high mobility group AT‐hook 1 promotes the binding of activating transcription factor 4 to the SLC7A11 promoter, thereby increasing SLC7A11 transcription and contributing to cisplatin resistance through ferroptosis suppression [41]. In addition, promoter–enhancer interactions regulate global lipid metabolic reprogramming associated with trastuzumab resistance [192].

NcRNAs are a class of functional transcripts that lack protein‐coding potential. Among the ncRNA family, miRNAs, long noncoding RNAs (lncRNAs), and circular RNAs (circRNAs) have been extensively studied for their roles in drug resistance–associated metabolic reprogramming in tumors. MiRNAs are small ncRNAs that regulate gene expression posttranscriptionally by inducing mRNA degradation and blocking translation initiation [195]. MiRNAs produced by tumor cells can target key enzymes involved in glucose, lipid metabolism and oxidative stress, driving cancer cells toward a drug‐resistant metabolic phenotype [80, 196, 197]. The lncRNA are a heterogeneous group of ncRNAs with multiple functions including stabilizers, scaffolds, competing endogenous RNAs (ceRNAs) and posttranslational modifications [198]. The lncRNA DIO3OS stabilizes the mRNA of lactate dehydrogenase A (LDHA) by protecting the integrity of its 3′UTR and activates glycolytic metabolism in aromatase inhibitor resistant breast cancer cells [199]. The lncRNA CTSLP8 facilitates the binding of pyruvate kinase M2 (PKM2) to the promoter region of c‐Myc, thereby upregulating glycolysis [200]. The lncRNA ANRIL acts as a ceRNA of miR‐125a to relieve its repressive effect on the glycolytic enzyme enolase (ENO1) [201]. The lncRNA A LENOX promoted the association of the RAP2C GTPase with mitochondrial fission regulator dynamin‐related protein 1 (DRP1), increasing DRP1 S637 phosphorylation, mitochondrial fusion, and OXPHOS, conferring resistance to mitogen‐activated protein kinase (MAPK) inhibition in melanoma [202]. CircRNAs are single‐stranded, covalently closed RNA molecules. They can act as molecular sponges to antagonize the function of miRNAs. CircHIF1A in CRC upregulates HIF‐1α and overexpresses glucose transporter 1 (GLUT1) and LDHA to restrain cetuximab treatment by targeting miR‐361‐5p [203]. In glioma, circKIF4A sponges miR335‐5p and restores the expression of aldolase A, accelerating glycolysis rate and promoting TMZ resistance [204]. In addition, circRNA–protein interactions also represent a key mechanism in mediating metabolic rewiring. CircABCC4 enhances the interaction between PKM2 and karyopherin α2 to promote PKM2 nuclear translocation in CAFs, leading to the transcription of glycolysis‐related genes and oxaliplatin resistance in pancreatic cancer [128].

Current research seems to suggest that epigenetic mechanisms may play a broader role than genetic alterations in mediating metabolic reprogramming associated with anticancer drug resistance. As previously described, metabolic reprogramming can contribute to resistance by influencing epigenetic modifications. Conversely, epigenetic regulators can alter the transcription of metabolic enzymes or signaling molecules, thereby forming a metabolic–epigenetic feedback loop that reinforces resistance mechanisms.

### 5.3 Key Transcription Factor Regulation

A range of transcription factors act as central hubs, integrating intra‐ and extracellular signals to promote the adaptation of tumor cells to therapeutic stress. STAT proteins, which play a key role in orchestrating the highly dynamic metabolism, frequently take the lead in driving metabolic rewiring to help tumor cells resist therapeutic attacks. STAT3, one of the most prominent members of this family, can dysregulate mitochondrial metabolism and force cells to depend on glycolysis by upregulating the expression of glycolytic enzymes and GLUTs [161, 205]. FAO is another metabolic pathway regulated by STAT3 and can mediate chemoresistance in breast cancer [206]. Increased FAO can activate STAT3, which then elevates phospholipid synthesis and overcomes chemotherapy‐induced tumor cell apoptosis [22]. In regulating redox homeostasis, STAT3 activated by TMZ upregulates CYP3A5 to refine NAD⁺/NADH for the enhancement of mitochondrial functions and DDR, thereby conferring TMZ resistance [53]. In the regulation of ferroptosis, STAT3 binds to consensus DNA response elements in the promoters of the genes associated with negative ferroptosis regulation, including GPX4 and SLC7A11 and leads to 5‐FU resistance in gastric cancer [36]. Apart from STAT3, STAT2 is found to regulate lipid droplet metabolism and activate mitophagy in pazopanib resistance [48].

Other transcription factors, such as HIF‐1α, also transduce metabolic signal changes sensed by tumors—such as those involving mTOR—during glycolysis‐driven chemoresistance [207, 208]. GATA‐binding protein 3 (GATA3), Rel‐B and ATF4 are all transcription factors that regulate antioxidant systems and confer resistance to ferroptosis‐related drugs [34, 37, 41, 140].

Overall, genetic and transcriptional remodeling participate in the precise regulation of metabolism to favor tumor resistance. However, current research has focused largely on well‐characterized metabolic rewiring mechanisms involved in drug resistance, such as glycolysis and ferroptosis, while the drivers underlying other preferential metabolism‐dependent resistance phenotypes are still poorly understood. Therefore, further investigations are needed to identify druggable upstream drivers.

## 6 Metabolism‐Targeted Strategies to Overcome Drug Resistance

Since metabolic reprogramming affects the efficacy of a wide variety of drug therapies, metabolism‐targeting agents provide new directions for overcoming drug resistance. Therefore, this section focuses on small‐molecule inhibitors that have been frequently reported to target divergent metabolic pathways, emphasizing their effects against resistance to chemotherapies, targeted therapies, and immunotherapies (Table 3).

**TABLE 3:** Metabolism‐targeted small‐molecule inhibitors used in combination with existing therapies to overcome tumor drug resistance.

Metabolic pathway	Agent	Target	Existing resistant therapy	Phase	Indication	References
Aerobic glycolysis	WZB117	GLUT1	Imatinib	Preclinical	Gastrointestinal stromal tumor	[209]
			Paclitaxel	Preclinical	OC, oral squamous cell carcinoma	[210]
	3‐Bromopyruvate	HK2	Platinum drugs	Preclinical	Colon cancer	[211]
	Shikonin	PKM2	GEM	Preclinical	Pancreatic cancer	[212]
	Galloflavin	LDHA, LDHB	Cisplatin	Preclinical	Burkitt's lymphoma	[213]
Glutamine metabolism	V‐9302	SLC1A5	Cetuximab	Preclinical	HNSCC	[214]
			Anti‐PD‐L1 antibody	Preclinical	Lung cancer, colon cancer	[215]
	JU‐083	GLS	Anti‐PD‐L1 antibody	Preclinical	Prostate cancer, bladder cancer	[216]
	DON	Glutamine	MBTA	Preclinical	Pancreatic cancer	[217]
			Trametinib	Preclinical	Pancreatic cancer	[218]
			Vemurafenib	Preclinical	Melanoma	[219]
	DRP‐104	Glutamine	Trametinib	Preclinical	PDAC	[218]
			Anti‐PD‐1 antibody	Preclinical	Lung cancer	[220]
	CB‐839	GLS	Cabozantinib	II (NCT03428217)	Renal cell carcinoma	[221]
			5‐FU	I (NCT02861300)	CRC	[222]
			Panitumumab	I/II (NCT03263429)	CRC	[223]
Fatty acid metabolism	Cerulenin	FASN	Cisplatin	Preclinical	OC	[224]
	TVB‐3166	FASN	Taxane	Preclinical	Prostate cancer	[225, 226]
			GEM	Preclinical	Bladder cancer	[227]
	TVB‐2640	FASN	Anti‐PD‐L1 antibody	Preclinical	HCC	[228]
			Taxanes	I (NCT02223247)	NSCLC, OC, and breast cancer	[226]
			Bevacizumab	II (NCT03032484)	Astrocytoma	[229]
	Etomoxir	CPT1	TMZ	Preclinical	GBM	[230]
Redox homeostasis	Erastin	VDACs	Docetaxel	Preclinical	Prostate cancer	[231]
			Oxaliplatin, 5‐FU	Preclinical	Gastric cancer	[232]
	RSL3	GPX4	PARPi	Preclinical	BRCA1‐mutant breast cancer	[33]
	6‐AN	G6PDH	Erlotinib	Preclinical	Pancreatic cancer	[233]
	NCT‐503	PHGDH	Sorafenib, Regorafenib, Lenvatinib	Preclinical	HCC	[234]
			Bortezomib	Preclinical	MM	[235, 236]
			Erlotinib		NSCLC	[237]
Mitochondrial metabolism	IACS‐010759	Complex I	Gefitinib	Preclinical	NSCLC	[238]
			Palbociclib	Preclinical	TNBC, ER (+) breast cancer	[239, 240]
	CPI‐613	Complex I	Gemcitabine, cisplatin	Preclinical	Cholangiocarcinoma	[241]
			5‐FU	Preclinical	CRC	[242]
			Cytarabine and mitoxantrone	I, II (NCT01768897, NCT02484391)	AML	[243]
			Modified FOLFIRINOX (fluorouracil, oxaliplatin, irinotecan, and leucovorin)	III (NCT03504423)	mPC	[244]
	DCA	PDK	Sorafenib	Preclinical	HCC	[245]

Small molecule inhibitors that target cancer metabolic reprogramming were selected in combination with existing therapies to overcome tumor drug resistance.

Abbreviations: 5‐FU, 5‐fluorouracil; AML, acute myeloid leukemia; CPT1, carnitine palmitoyltransferase 1; CRC, colorectal cancer; ER, estrogen receptor; FASN, fatty acid synthase; G6PDH, glucose‐6‐phosphate dehydrogenase; GBM, glioblastoma multiforme; GEM, gemcitabine; GLS, glutaminase; GLUT1, glucose transporter 1; GPX4, glutathione peroxidase 4; HCC, hepatocellular carcinoma; HK2, hexokinase 2; HNSCC, head and neck squamous cell carcinoma; LDHA, lactate dehydrogenase A; LDHB, lactate dehydrogenase B; MBTA, anti‐CD40 antibody; MM, multiple myeloma; mPC, metastatic pancreatic adenocarcinoma; NSCLC, non‐small cell lung cancer; OC, ovarian cancer; PARPi, poly (ADP‐ribose) polymerase inhibitor; PDAC, pancreatic ductal adenocarcinoma; PDK, pyruvate dehydrogenase kinase; PHGDH, phosphoglycerate dehydrogenase; PKM2, pyruvate kinase M2; TMZ, temozolomide; TNBC, triple‐negative breast cancer; VDACs, voltage‐dependent anion channels.

### 6.1 Targeting Nutrient Utilization

Tumor cells develop heterogeneous metabolic preferences distinct from those of normal tissues. Therefore, selectively targeting the nutrient dependencies of cancer cells holds promise for overcoming drug resistance. In this section, we introduce small‐molecule inhibitors that target tumor utilization of glucose, amino acids, and lipids.

#### 6.1.1 Aerobic Glycolysis

The first requirement for glycolysis is the uptake of adequate amounts of glucose via glucose transporters. Glucose transporter (GLUT), which is known to be overexpressed in various cancers, plays a crucial role in this process. WZB117 is a GLUT1 inhibitor that exerts excellent antitumor effects on different cancers [246]. WZB117 can significantly suppress BCL‐2 expression, inducing apoptosis in imatinib‐resistant and paclitaxel‐resistant cancers [209, 247]. SMI277 is another new GLUT1 inhibitor with stronger inhibitory effects on glucose uptake than WZB117 [248]. However, its potential role in overcoming drug resistance warrants further investigation. Since none of these GLUT inhibitors have progressed to clinical research, whether they can exert antiresistance effects in patients remains to be determined.

Small‐molecule inhibitors that target glycolysis also are involved in the inhibition of enzymes, such as HK, PK, and LDH. HK2, the isoform of HK that phosphorylates glucose as the first key enzyme in glycolysis, is highly expressed in cancers [221]. Among HK2 inhibitors, 3‐bromopyruvate (BP) has superior toxic effects on HCC [210], CRC [249], OC [250], and thyroid cancer [251]. One study identified BP as a potential agent that increases the chemosensitivity of resistant p53‐deficient cells to platinum drugs [211]. PK is the pivotal enzyme in the last rate‐limiting step of glycolysis, and it catalyzes the transformation of phosphoenolpyruvate to pyruvate, which is an energy regeneration process that occurs independently of the oxygen supply. Among the isoforms, PKM2 is expressed at high levels in cells undergoing rapid nucleic acid synthesis. Shikonin, a bioactive compound derived from traditional Chinese medicine with broad pharmacological effects, exerts some of its activity through the inhibition of PKM2 and reverses GEM resistance in pancreatic cancer cells [212]. However, in head and neck squamous cell carcinoma (HNSCC), the PKM2 activator DASA‐58 exerts cytotoxic effects on cell survival, and some studies have demonstrated that decreased PKM2 expression can induce tumor growth [221, 252]. Therefore, the scope of the application of PKM2 inhibitors needs further investigation.

The reciprocal conversion of pyruvate and lactate is catalyzed by LDH. Specifically, LDHA predominantly catalyzes the conversion of pyruvate to lactate, which is a crucial step in the Warburg effect. Conversely, LDHB facilitates the reverse reaction, enabling tumor cells to utilize lactate from the TME [253]. Hence, the inhibition of both LDHA and LDHB is necessary. Galloflavin is a dual‐targeting inhibitor that exhibits threefold greater selectivity for LDHA than for LDHB [254]. Its inhibition of lactate production enhances and activates tumor‐infiltrating CD8⁺ T cells, thereby improving the efficacy of anti‐PD‐1 therapy [255]. In addition, the combination of galloflavin to treat cisplatin‐resistant Burkitt's lymphoma has potential of the drug for overcoming resistance [213].

#### 6.1.2 Glutamine Metabolism

The mechanisms of glutamine metabolism inhibitors include inhibiting the glutamine transporter, SLC1A5, inhibiting related metabolic enzymes, GLS, and competitively antagonizing glutamine. By competitively antagonizing SLC1A5, the small molecule inhibitor V‐9302 can inhibit glutamine flux and improve the toxicity of cetuximab in HNSCC [214]. In addition, V‐9302 plays a critical role in immunotherapy. When the uptake of glutamine by tumor cells is inhibited by the utility of V‐9302, CD8+ T cells gain increased access to glutamine to support their effector functions, thereby contributing to the reversal of resistance to anti‐PD‐L1 antibody therapy [215, 256]. However, V‐9302 has poor aqueous solubility, and tumor cells might shift to a glucose metabolism‐dependent state during glutamine inhibition [257].

The key enzyme in glutaminolysis, GLS, converts glutamine to glutamate for the subsequent TCA cycle. The GLS inhibitor, JU‐083 reprograms the metabolic phenotype of TAMs toward increased glycolysis and a disrupted TCA cycle, promoting antitumor immune responses [216]. However, prolonged inhibition by JHU‐083 upregulated PD‐L1 expression in bladder cancer cells. Combination treatment with JHU083 and gefitinib reverses the PD‐L1 upregulation, thereby alleviating T cell immunosuppression and significantly improving therapeutic outcomes [258].

6‐Diazo‐5‐oxo‐l‐norleucine (DON) is an effective glutamine antagonist with significant potential. DON has shown remarkable efficacy in immunotherapies. In murine models with subcutaneous Panc02 tumors, DON guaranteed the efficacy of an anti‐CD40 antibody (MBTA), and complete tumor elimination was achieved in half of the treated animals [217]. Among the targeted therapies available, DON prevents resistance to MAPK and ERK (extracellular signal‐regulated kinase) inhibitors [218, 219]. DRP‐104, the prodrug of DON, can also overcome resistance to immunotherapy and ERK inhibitors but can accumulate in tumor tissues more accurately than DON can [218, 220, 259, 260].

#### 6.1.3 FA Metabolism

The regulation of FA biosynthesis and oxidation is also a valuable target. The inhibition of FASN in cancer has been studied for decades, leading to the development of various potent inhibitors. Cerulenin is the first FASN inhibitor and has been shown to reverse platinum resistance in OC cells [224]. Further study revealed that cerulenin‐induced apoptosis is mediated by topoisomerase I, which increases intracellular polyunsaturation; therefore, combination therapy comprising cerulenin and the topoisomerase I suppressor LY294002 has synergistic effects [261]. TVBs, which are novel potent FASN inhibitors, are currently being studied for their ability to reverse resistance to drugs including taxanes, GEM and PD‐L1 checkpoint blockade [225, 227, 228]. Taken together, these findings suggest that targeting FASN is a promising research direction for inhibiting de novo FA synthesis.

Etomoxir, which is a CPT1 inhibitor, decreases the stemness and invasiveness of GBM cells, restores the toxicity of TMZ and prolongs survival outcomes in vivo [230]. CPT1 inhibition can also promote an antitumor immune microenvironment alongside HER2‐targeted therapies to combat resistance in HER2+ breast cancer patients. However, CPT1‐deficient cells exhibit increased glucose dependency, which enables survival. Therefore, compensatory pathways also need to be simultaneously inhibited [262].

### 6.2 Disrupting Redox Homeostasis

Since many metabolism‐related mechanisms of drug resistance stem from the ability of cancer cells to counteract drug‐induced oxidative stress, agents that target redox homeostasis can help overcome such resistance. Erastin, one of the most classic ferroptosis inducers, activates voltage‐dependent anion channels (VDACs), leading to increased enhanced ROS production. In addition, erastin lowers GSH levels by inhibiting system Xc^−^. The administration of erastin ensures sufficient cytotoxic ROS production to reverse chemoresistance [231, 232]. Inhibitors of ferroptosis defense system, such as the GPX4 inhibitor (GPX4i) RSL3, can also accumulate ROS for ferroptosis. Specifically, BRCA1 promotes the transcription of voltage‐dependent anion channel 3 and GPX4. As a result, BRCA1‐mutant cells resistant to PARP inhibitors are resistant to erastin‐induced ferroptosis but remain sensitive to GPX4i [33].

Targeting the PPP represents another strategy to suppress ROS, by decreasing the cellular NADPH/NADP⁺ ratio. 6‐Aminonicotinamide (6‐AN) is a competitive inhibitor of G6PDH and therefore blocks glucose entry into the PPP. In drug‐resistant pancreatic cancer, treatment with 6‐AN induces G1 cell cycle arrest and restores sensitivity to erlotinib [233].

SSP also contributes to the production of the antioxidants GSH and NADPH [263]. PHGDH is the first rate‐limiting enzyme in the SSP. The PHGDH inhibitor, NCT‐503 is a widely used piperazine‐1‐thiourea‐based small molecule inhibitor [264]. It has been demonstrated to ameliorate ROS levels, overcoming resistance to TKIs, including sorafenib, regorafenib and lenvatinib, in HCC; erlotinib in NSCLC; and the proteasome inhibitor bortezomib in multiple myeloma [234, 235, 236, 237]. However, further investigation into their kinetics and toxicity is necessary, as these inhibitors have not yet undergone human clinical trials.

### 6.3 Modulating Mitochondrial Metabolism

Either inhibiting or promoting mitochondrial metabolism have shown promising potential for overcoming tumor drug resistance. In the ETC of mitochondria, which is composed of 4 complexes, complex I (NADH‐coenzyme Q oxidoreductase) transfers electrons from NADH to ubiquinone, producing a proton gradient across the inner mitochondrial membrane that is crucial for ATP synthesis. Biguanides, which are primarily used for the treatment of diabetes, are currently a hot topic in cancer research because of their ability to target complex I and inhibit OXPHOS. However, few studies have revealed their ability to prevent drug resistance. In addition to biguanides, some nonbiguanide small molecule inhibitors, such as IACS‐010759, also exhibit a high degree of selectivity for inhibiting complex I [265]. Currently, the role of IACS‐010759 in OXPHOS‐dependent tumors is being studied. In NSCLC, epigenetic alterations increase the activation of OXPHOS, leading to acquired EGFR‐TKI resistance, and IACS‐010759 successfully reversed gefitinib resistance in a patient‐derived xenograft model in vivo [238]. Moreover, both triple‐negative breast cancer and ER+ breast cancer cells exhibited decreased resistance to palbociclib when treated with IACS‐010759 [239, 240]. However, a phase I trial examining the safety, tolerability and tolerable dose of IACS‐010759 revealed a narrow therapeutic range (NCT02882321). This limitation poses challenges in maintaining targeted exposure and may lead to dose‐limiting toxicities, including increased blood lactate levels and neurotoxicity [266]. The nonspecific mitochondrial inhibitor CPI‐613 is a lipoate analog and can inhibit lipoate‐dependent mitochondrial enzymes (PDH and α‐ketoglutarate dehydrogenase) [267]. Combination therapy with CPI‐613 can restore the efficacy of various chemotherapeutic agents, including 5‐FU, irinotecan, GEM, and cisplatin [241, 242]. Cotreatment with the GLS1 inhibitor CB‐839 in CPI‐613 treatment eliminates the glutamine metabolic dependency induced by the TCA cycle inhibition [268]. However, further preclinical and clinical studies are necessary to decrease the side effects of drugs, broaden their application and develop precision treatments.

Instead of directly and lethally inhibiting respiratory function, reversing aerobic glycolysis and facilitating the entry of pyruvate into mitochondria constitute an alternative strategy for combatting drug resistance in cancer. PDH kinase (PDK) negatively regulates PDH. Hence, inhibition of PDK alters aberrant glucose metabolism and restores OXPHOS and oxidant reactions in tumor cells. Dichloroacetate (DCA) is an interesting small molecule that was first utilized in some metabolic disorders, including diabetes mellitus, lipid and lipoprotein disorders, and lactic acidosis [269]. In recent years, DCA has been repurposed for cancer treatment since it can effectively inhibit PDK activity. In HCC, DCA effectively converts metabolic activity from glycolysis to OXPHOS, eliminating factors that cause to sorafenib resistance [245].

### 6.4 Combinatorial Approaches in Clinical Trials

Given the promising ability of metabolism‐targeting agents to overcome drug resistance in experimental models, numerous clinical trials are currently in progress to evaluate their therapeutic potential. These agents are being integrated into existing therapeutic regimens including chemotherapy, targeted therapy, and immunotherapy. For chemotherapy resistance, cytarabine and mitoxantrone combined with CPI‐613 have been evaluated in phase I/II trials for AML (NCT01768897, NCT02484391), with a 54% response rate in phase I. In phase II, a dose‐response trend was observed in older but not younger patients, potentially linked to age‐related declines in mitochondrial function and autophagy, although this finding requires further validation [243]. Modified FOLFIRINOX (fluorouracil, oxaliplatin, irinotecan, and leucovorin), a first‐line regimen for metastatic pancreatic adenocarcinoma (mPC), was also combined with CPI‐613 in a randomized phase III trial, but failed to improve prognosis in both short‐ and long‐term mPC patients (NCT03504423) [244]. A study investigating CPI‐613 in combination with HCQ and either 5‐FU or GEM for advanced refractory solid tumors is currently ongoing (NCT05733000). Other chemotherapy regimens, such as 5‐FU combined with the GLS inhibitor CB‐839 in PIK3CA‐mutant CRC, and taxanes combined with FASN inhibitor TVB‐2640, have both completed phase I trials with promising outcomes (NCT02861300, NCT02223247) [222, 226]. For targeted therapy resistance, in a randomized phase II study, the combination of bevacizumab and TVB‐2640 demonstrated statistically significant improvement over historical bevacizumab monotherapy, with a favorable safety profile (NCT03032484) [229]. Panitumumab combined with CB‐839 has also shown safety and promising preliminary responses in a phase I/II study of metastatic CRC (NCT03263429) [223]. To overcome resistance to immunotherapy, multiple research groups are initiating clinical studies of DRP104 in fibrolamellar HCC (FLC) and other advanced solid tumors (NCT06027086, NCT04471415).

The use of metabolism‐targeting agents does not perfectly overcome tumor resistance, as ideally expected. Therefore, it's necessary to perform metabolic profiling of tumors for different organs and subtypes and identify corresponding detectable tumor biomarkers since the metabolic pathways supporting the survival of different tumor cells vary significantly, and this classification aids not only in precision and personalized therapy but also in preventing the resistance to metabolism‐targeting therapies.

## 7 Clinical Challenges and Future Perspectives

Metabolic reprogramming is increasingly recognized as a key hallmark of drug‐resistant tumor cells. However, monotherapy with metabolism‐targeting agents is often ineffective against drug‐resistant tumors, and clinical studies combining these agents with existing therapies are still at an early stage, indicating a long and challenging road ahead. Therefore, given the complexity of the interplay between metabolism and drug resistance, there is still much to explore in this area. First of all, drug‐resistant tumor cells may rely on multiple reprogrammed metabolic pathways; therefore, therapeutic strategies should aim to identify and target their most vulnerable metabolic dependencies. In addition, metabolic reprogramming not only provides energy and synthetic materials for tumor cells to resist drugs but also supplies materials for processes such as signal transduction, epigenetic modifications, and posttranslational modifications of proteins. However, our exploration of the latter has just begun. Only with a clear understanding of the functions of metabolic products can targeted clinical strategies be developed to overcome drug resistance. Moreover, tumors exhibit profound metabolic plasticity and compensatory reprogramming, whereby the inhibition of one metabolic pathway induces the overactivation of alternative routes, ensuring the continued survival of drug‐resistant cells. Hence, in addition to combining basic treatments, combination therapies that include different metabolic inhibitors need further consideration.

Further investigation into the upstream regulatory mechanisms is also essential. However, existing studies have yet to comprehensively cover all forms of metabolic reprogramming, which leaves a substantial gap in the identification of reliable biomarkers for resistant tumors amenable to targeted metabolic therapies. In addition, although current studies highlight transcriptional and posttranscriptional regulation as the predominant modes of controlling metabolic reprogramming, aberrations at the genetic level also need investigation to better correlate genotypes with metabolism‐associated drug resistance, thereby guiding clinical therapy.

In addition to tumor cells themselves, metabolic crosstalk within the TME should not be overlooked. Metabolic reprogramming of both tumor cells and immune cells influences antitumor immunity and responsiveness to immune checkpoint inhibitors; however, the application of metabolic interventions in this context remains limited. Amino acid metabolism plays a critical role in shaping antitumor immune responses, yet targeted therapies against amino acid metabolism are still underdeveloped.

In conclusion, metabolic reprogramming is a nonnegligible contributor to drug resistance, and the underlying mechanisms need to be explored further. According to research on metabolism in drug‐resistant tumors, metabolic subtyping of tumors can be performed, allowing for more precise guidance in clinical treatment. The development of more effective small‐molecule inhibitors that target metabolism is urgently needed since the number of inhibitors available for clinical use is limited. All these progresses will ultimately provide better solutions for addressing the challenges of drug resistance in clinical cancer therapy.

## Author Contributions

Conceptualization: J.‐Y.F., R.‐B.J., and X.‐Y.W. Writing—original draft: Y.‐H.Z., W.‐J.Y., and L.‐F. T. Writing—review and editing: Y.‐H.Z., W.‐J.Y., and L.‐F.T. Supervision: S‐F.G., J.‐Y.F., R.‐B.J., and X.‐Y.W. Funding acquisition: R.‐B.J., and X.‐Y.W. All authors have read and approved the final manuscript.

## Ethics Statement

The authors have nothing to report.

## Conflicts of Interest

The authors declare no conflicts of interest.

## Data Availability

The authors have nothing to report.
